# CdS quantum dots modified CuO inverse opal electrodes for ultrasensitive electrochemical and photoelectrochemical biosensor

**DOI:** 10.1038/srep10838

**Published:** 2015-06-04

**Authors:** Lei Xia, Lin Xu, Jian Song, Ru Xu, Dali Liu, Biao Dong, Hongwei Song

**Affiliations:** 1 State Key Laboratory on Integrated Optoelectronics, College of Electronic Science and Engineering, Jilin University, 2699 Qianjin Street, Changchun 130012, China

## Abstract

The CuO inverse opal photonic crystals (IOPCs) were synthesized by the sol-gel method and modified with CdS quantum dots by successive ionic layer adsorption and reaction (SILAR). CdS QDs modified CuO IOPCs FTO electrodes of different SILAR cycles were fabricated and their electrochemical properties were studied by cyclic voltammetry (CV) and chronoamperometry (I–t). Structure and morphology of the samples were characterized by transmission electron microscopy (TEM), scanning electron microscopy (SEM), high-resolution TEM (HRTEM), Energy-dispersive X-ray analysis (EDX) and X-ray diffraction pattern (XRD). The result indicated that the structure of IOPCs and loading of CdS QDs could greatly improve the electrochemical properties. Three SILAR cycles of CdS QDs sensitization was the optimum condition for preparing electrodes, it exhibited a sensitivity of 4345 μA mM^-1^ cm^-2^ to glucose with a 0.15 μM detection limit (S/N= 3) and a linear range from 0.15 μM to 0.5 mM under a working potential of +0.7 V. It also showed strong stability, good reproducibility, excellent selectivity and fast amperometric response. This work provides a promising approach for realizing excellent photoelectrochemical nonenzymatic glucose biosensor of similar composite structure.

Determination of glucose with the help of glucose biosensors is a widely investigated and well developed field, due to its various important applications in clinical detection, biological analysis, environmental monitoring and food industry^1^,^2^,^3^. The most commonly used glucose biosensors are electrochemical enzyme based sensors, such as the glucose oxidase biosensor. However, these enzyme based biosensors have several drawbacks including the high cost of enzymes, lack of chemical and thermal stability, complicated procedures for enzyme immobilization, careful control of temperature and pH conditions, as well as poor reproducibility^4^,^5^,^6^,^7^. To overcome such limitations, glucose biosensors are increasingly being prepared without enzymes. In recent years, most of the research on these non-enzymatic electrochemical glucose biosensors has focused on using noble metals^8^,^9^,^10^,^11^, metal nanoparticles^12^,^13^,^14^, metal oxides^15^,^16^,^17^ and boronic acid^18^,^19^,^20^,^21^,^22^ as electrodes (anodes) for the detection of glucose.

Some metal oxides are being increasingly used in non-enzymatic glucose biosensor applications due to their low cost, excellent electrochemical properties and higher sensitivity. Among these materials, one of the most highly investigated oxides is copper oxide CuO, which is a p-type semiconductor with a narrow band gap of 1.34 eV. Its importance in the field of non-enzymatic glucose sensing can be attributed to its ease of synthesis, good chemical stability, electrocatalytic property and outstanding redox behavior. Hence, CuO has found wide applications and is commonly used in the preparation of catalysts, gas sensors, biosensors, semiconductors and Li-ion rechargeable batteries^23^,^24^,^25^,^26^.

Apart from the redox properties, the structure of the metal electrode is also important as it can affect the electrochemical behavior of the material. It is known that electrodes with a well-designed nanostructure are likely to have improved electrochemical behavior and performance, due to the increased surface area, improved electron transport, reduced minority-carrier diffusion length and lower electron–hole recombination loss in nanostructures^27^,^28^. In particular, regularly ordered nanostructures, such as nanoflowers^29^, nanorods^30^, nanospheres^31^, and nanowires^32^,^33^, have been demonstrated to significantly enhance electron transport, resulting in excellent electrochemistry properties. However, there is still further room for improvement in this field to develop new and better metal structures for optimal electrochemical properties.

One such newly developed material is the Inverse Opal Photonic Crystal (IOPC), which is a replicated shell structure of a face-centered-cubic (fcc) opal. The IOPC structure offers very high specific surface area and porosity (74% void volume). In addition, the slow light effect caused by the structure of periodical 3D IOPCs can enhance the sensitivity in photoelectrochemical detection^34^. However, to the best of our knowledge, no research has been reported to date on the use of CuO IOPCs as photoanodes in glucose sensors.

In this work, CuO IOPCs have been synthesized for the first time as electrochemical and photoelectrochemical biosensors. The structure of IOPCs provides large surface area and uniform porous distribution, which are conducive to increased contact with glucose solution and better electronic transmission. The large surface area provides more sites for loading other materials and allows intimate contact with the electrolyte. The porous structure may also shorten the electronic diffusion distance between the Fluorine-doped tin oxide (FTO) glass electrode substrate and the redox centers, thus improving the electron transfer ability^35^,^36^.

It should be highlighted that CuO mainly absorbs infrared light as a narrow-band semiconductor. In order to obtain visible light absorption and better photoelectrochemical properties, we further modified the CuO IOPCs by incorporating cadmium sulfide (CdS) quantum dots (QDs) on the electrode surface.

Cadmium sulfide, one of the most technologically important semiconductor materials, has applications in a wide range of fields including optoelectronics^37^, photovoltaics^38^, chemical sensors^39^, and photocatalysis^40^. With a band gap of around 2.4 eV, which matches well with the visible spectral range of solar irradiation, CdS exhibits excellent photocatalytic activity because of its highly effective absorption of solar energy.

The CuO IOPCs were incorporated with cadmium sulfide (CdS) quantum dots by the direct adsorption of ions and subsequent chemical precipitation, and this technique is referred to as Successive Ionic Layer Adsorption and Reaction (SILAR). The method involves immersion of CuO IOPCs electrodes in solutions of Cd^2+^ and S^2-^ successively to deposit CdS nanocrystallites on the surface of CuO IOPCs electrodes. This type of solution–based deposition of CdS and CdSe quantum dots on metal oxide surfaces, similar to SILAR, has already been successfully employed by Hodes and coworkers^41^,^42^.

In this work, we report the preparation, characterization and properties of a novel and innovative electrode design which involves the beneficial combination of CuO IOPCs with CdS QDs sensitization.

## Results and Discussion

### Material Characterization

**Figure 1:**
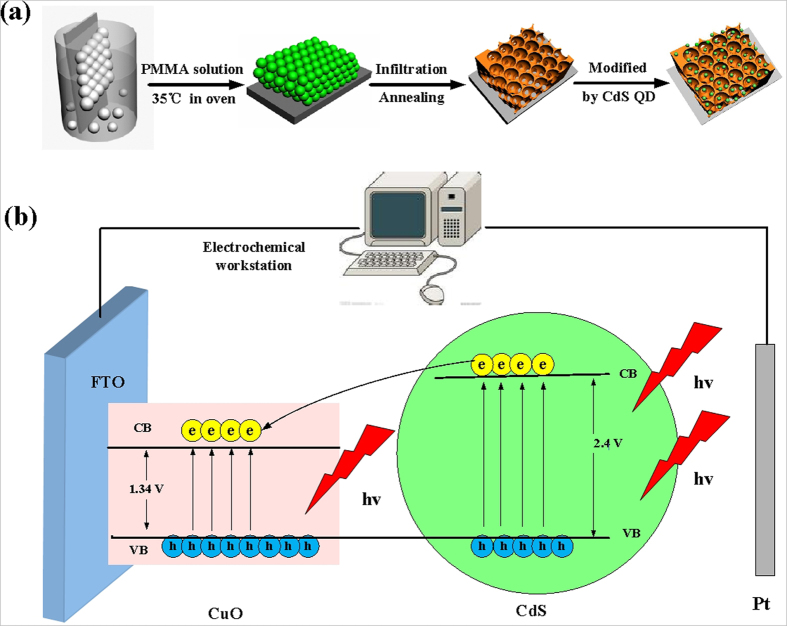
(**a**) Schematic illustration for the synthesis of CdS quantum dots modified CuO IOPCs FTO electrodes. (**b**) Schematic illustration for the reaction process of CdS quantum dots modified CuO IOPCs FTO electrodes with Xe-lamp illuminating.

The experimental procedure for the preparation of CdS QDs modified CuO IOPCs FTO electrodes consists of four simple steps, as illustrated in Fig. 1a: 1) self-assembly of close-packed polymethyl methacrylate (PMMA) spheres with multilayer structures onto FTO substrates, 2) infiltration of PMMA template with prepared precursor solution of CuO by sol-gel method, 3) removal of the original PMMA template by high temperature annealing to obtain CuO IOPCs, and 4) final sensitization of the CuO IOPCs with CdS QDs using SILAR route.

**Figure 2:**
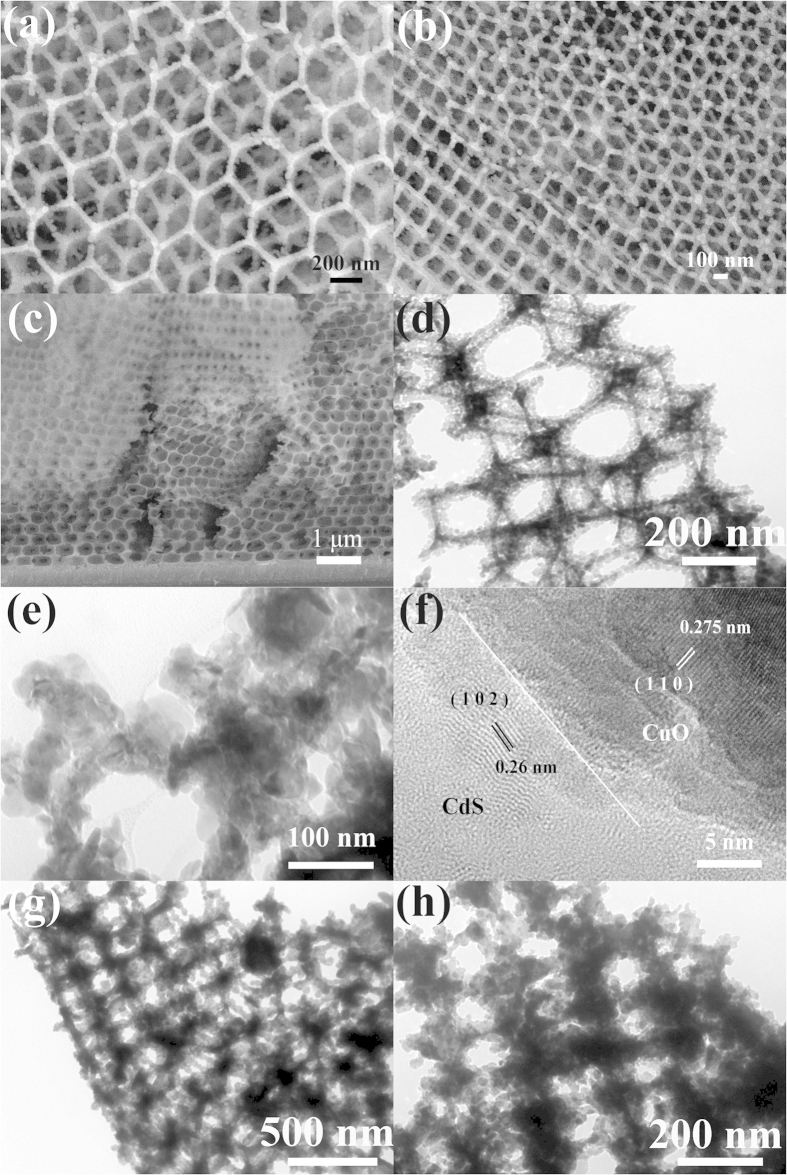
(**a**) - (**b**) SEM image of CuO IOPCs S1 and S2. (**c**) SEM images of cross-section view of CuO IOPCs. (**d**) TEM image of CuO IOPCs S1. (**e**) TEM image of CdS QDs modified CuO IOPCs of 2 SILAR cycles. (**f**) HRTEM image of the CdS/CuO interface. (**g**) - (**h**) TEM image of CdS QDs modified CuO IOPCs of 3 and 5 SILAR cycles.

Characterization of the surface morphology and microstructure of the synthesized CuO IOPCs were performed by field emission scanning electron microscope (SEM) and transmission electron microscopy (TEM). The SEM images of the synthesized CuO IOPCs (Fig. 2a,b) show a long-range ordered hexagonal close-packed arrangement of the inverse opal structure in three-dimensional space. Two variants were observed, with diameters of 378 nm and 275 nm, and they were named as CuO IOPCs S1 and CuO IOPCs S2 respectively. As shown in Fig. 2c, the cross-sectional SEM images of CuO IOPCs also displayed a vertical ordering with face-centered-cubic (fcc) packing structure. In order to investigate further into the detailed structure and morphology of CuO IOPCs, high-magnification TEM images were obtained. The TEM image in Fig.2d illustrates that the wall thickness of the CuO IOPCs is about 45 nm, and shows that the structure consists of a large amount of small nanoparticles (NPs) and neighboring pores that are connected to each other by the thin wall. The small wall thickness of the CuO IOPCs structure is highly conductive to the unidirectional transmission of electrons. The TEM image in Fig. 2e shows that the inner and outer surfaces of the CuO IOPCs are well covered with small CdS quantum dots after 2 SILAR cycles. This coverage amount indicates that the Cd^2+^ and S^2–^ ions are able to easily diffuse into the pores of the IOPCs and form CdS nanocrystallites. Good crystalline structures were observed for both the CuO and CdS materials, from the high-resolution TEM (HRTEM) image (Fig. 2f). The thickness of the CdS layer was found to be around 5 nm. Interplanar distances of the fringes of the CuO and CdS QDs are 0.275 nm and 0.26 nm, which correspond to the *d* spacing of (110) planes of monoclinic CuO and the (102) planes of hexagonal CdS, respectively.

**Figure 3:**
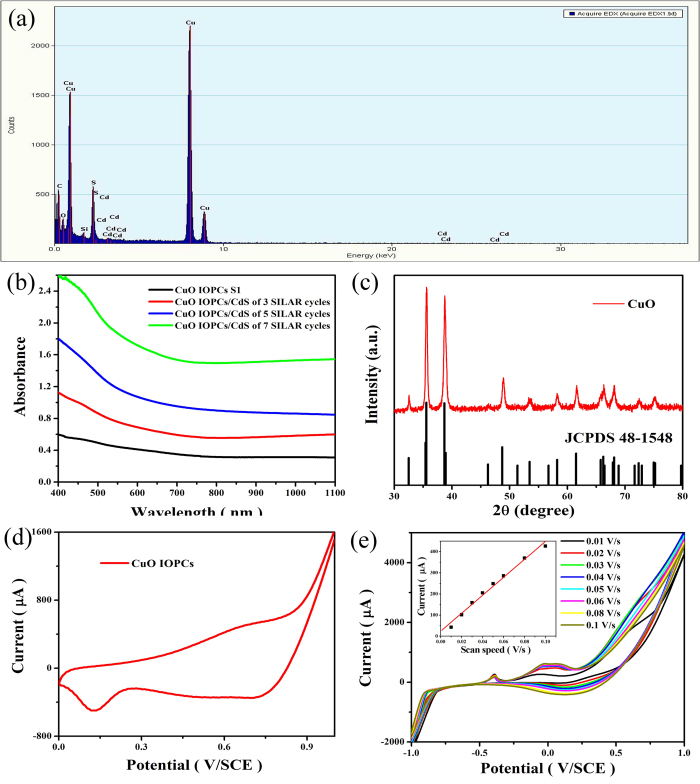
(**a**) EDX analysis of CdS QDs modified CuO IOPCs of 2 SILAR cycles. (**b**) Absorption spectra of different electrodes: CuO IOPCs S1 (black), CdS QDs modified CuO IOPCs of 3, 5, 7 SILAR cycles (red, blue, green). (**c**) XRD patterns of CuO IOPCs. (**e**) Cyclic voltammograms of CuO IOPCs FTO electrode in 0.1 M NaOH. (**e**) Cyclic voltammograms of CdS QDs modified CuO IOPCs FTO electrode at different scan rates. (Inset) Plots of peak currents versus scan rate.

To analyze the elemental components of the compound, Energy-dispersive X-ray analysis (EDX) was performed, and it was found that the compound consists of Cd and S, besides Cu and O (Fig. 3a), as expected. The lattice information and EDX analysis results confirm that the CdS QDs modified CuO IOPCs composites were successfully synthesized.

In order to assess the CdS QDs deposition and growth process on the CuO IOPCs, the TEM images and absorption spectra were monitored at different stages of SILAR route. Fig. 2g,h represent the TEM images of CdS QDs modified CuO IOPCs after 3 and 5 SILAR cycles, respectively. It can be seen from Fig. 2g that the structure of the CuO IOPCs remains intact and the CdS QDs cover the skeleton surface of the IOPCs after 3 SILAR cycles. With increasing number of SILAR cycles, the CdS QDs were deposited not only on the surface of the skeleton but also within the pores of the CuO IOPCs structure (Fig. 2h). The excess CdS QDs deposited in the pores could decrease the surface area and affect the contact of CuO with electrolyte. As seen from the absorption spectra (Fig. 3b), the absorption intensity increases with higher number of SILAR cycles. Also, the edge of the absorption band has a slight redshift from 457 nm to 482 nm (Figure S1), which can be attributed to the increased size and decreased bandgap of QDs^43^.

The phase properties of the synthesized CuO IOPCs were investigated by X-ray powder diffraction (XRD) experiments. As shown in Fig. 3c, the XRD patterns of the CuO IOPCs matched well to the hexagonal CuO phase (JCPDS, card no: 48-1548), which suggests that the monoclinic CuO phase was formed during the process of heat treatment. No characteristic impurity peaks were observed, indicating a pure monoclinic CuO phase in the samples.

### Electrochemical Properties of CuO IOPCs and CdS QDs modified CuO IOPCs FTO Electrode

The key parameters of the electrochemical kinetics of CuO IOPCs were obtained from cyclic voltammetry (CV) experiments performed in 0.1 M NaOH solution at a scan rate of 100 mV s^-1^. As seen from the CV curves in Fig. 3d, oxidation and reduction peaks were obtained at potentials of approximately 0.66 V and 0.7 V respectively, versus a saturated calomel electrode reference electrode. These peaks are believed to correspond to the Cu(II)/Cu(III) redox couple, which is the key component for non-enzymatic electrochemical glucose detection, according to previous studies^17^. The corresponding reaction processes are,


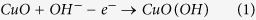



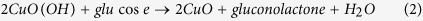


The effect of scan rate on the electrochemical response of CdS QDs/CuO IOPCs modified FTO electrode was investigated, and the results are shown in Fig. 3e. The measurement was performed in 0.1 M NaOH solution at different scan rates of 10-100 mV/s after 3 mM glucose injection. The redox peak currents show a linear response to the scan rate, indicating that the electron transfer process in the CuO redox reaction takes place through a surface-controlled mechanism^44^.

### Amperometric Detection of the Electrochemical and Photoelectrochemical biosensor

Current−time (I−t) curves were obtained to determine the amperometric sensing property of CuO IOPCs and CdS QDs modified CuO IOPCs FTO electrodes. All measurements were taken in 0.1 M NaOH solution at an applied potential of +0.7 V.

**Figure 4:**
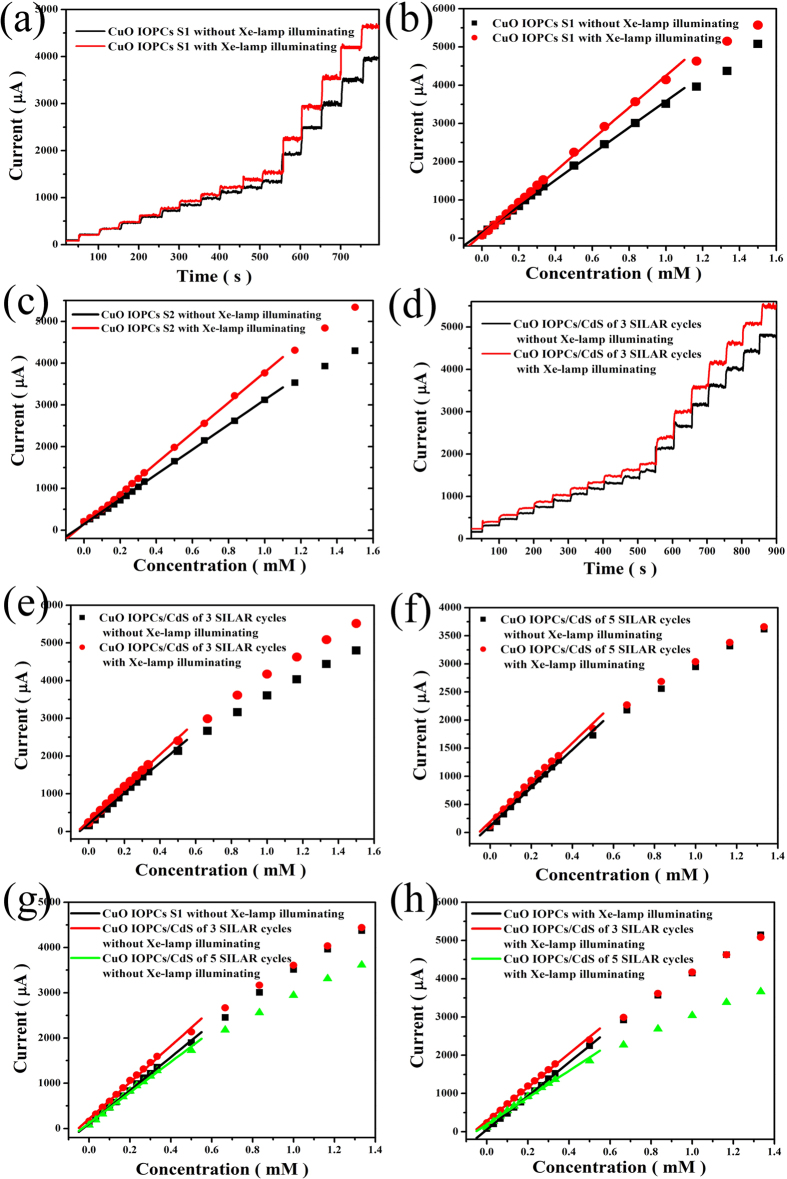
(**a**) Amperometric response of CuO IOPCs S1 with Xe-lamp illuminating and without Xe-lamp illuminating to successive addition of glucose at an applied potential of 0.7 V. (**b**) The corresponding calibration curves of (**a**). (**c**) Calibration curves of amperometric response of CuO IOPCs S2. (**d**) Amperometric response of CdS QDs modified CuO IOPCs of 3 SILAR cycles. (**e**) The corresponding calibration curves of (**d**). (**f**) Calibration curves of amperometric response of CdS QDs modified CuO IOPCs of 5 SILAR cycles. (**g**) - (**h**) Calibration curves of amperometric response of three different electrodes without Xe-lamp illuminating and with Xe-lamp illuminating: CuO IOPCs S1 (black), CdS QDs modified CuO IOPCs of 3, 5 SILAR cycles (red, green).

Figure 4a displays the typical amperometric responses of CuO IOPCs S1 photoelectrochemical sensor with successive addition of glucose, both with and without Xe-lamp illumination. The amperometric response indicates that the CuO IOPCs modified FTO electrodes have a fast electrochemical response of less than 5 s to the glucose. Figure 4b shows the corresponding calibration curves. Linear relationships were obtained between the current response and glucose concentration, both with and without Xe-lamp illumination. From the slope of the lines, sensitivity values were determined. The sensitivity was found to be 4065 μA mM^-1^ cm^-2^ with Xe-lamp illumination, which was about 1.2 times higher than the sensitivity without Xe-lamp illumination (3417 μA mM^-1^ cm^-2^). This difference indicates that Xe-lamp illumination improves the sensitivity of the CuO IOPCs electrodes.

The enhancement of sensitivity in the presence of Xe-lamp illumination can be attributed to the following two reasons. First, since the CuO electronic band gap is around 1.34 eV, corresponding to a wavelength of around 925 nm, the presence of light with wavelength less than 925 nm (higher energy) can cause the electrons to absorb photons and move from the valance band to the conduction band. Thus, when the electrodes are illuminated with Xe-lamp, more number of electrons are generated on the surface of the CuO IOPCs, which then participate in the redox reaction, as shown in equation 1. Second, the generation of a new electrical field inside the CuO nanoparticles due to the Xe-lamp illumination, inhibits the reverse direction recombination of electrons and holes, thus leading to improved sensitivity.

In order to demonstrate the advantages of the IOPCs structure, a reference CuO thin film FTO electrode was prepared by a spin-coating method from the same precursor solution of CuO as that used for preparing the Cuo IOPCs, and then annealed under the same condition. The electrochemical response calibration curves of the resulting reference thin film electrode are displayed in Figure S2. The linear response range of the reference thin film electrode was found to be up to 500 μM, while the linear response range of the IOPCs electrode was up to 1000 μM. This difference is likely due to the larger surface area of IOPCs, which allows more CuO nanoparticles to come in contact with the glucose solution before reaching saturation. The thickness of the CuO IOPCs and thin film electrodes were found to be 26 μm and 70 μm, respectively, as shown in Figure S3a and S3b. Moreover, the large void volume in the IOPCs structure (74%) makes the IOPCs lower in quality compared to the thin film electrode. However, the sensitivity of the reference thin film electrode was 1920 μA mM^-1^ cm^-2^, which is significantly lower than that of the IOPCs electrode (3417 μA mM^-1^ cm^-2^) without Xe-lamp illumination. There are two reasons for this observed difference: lower surface area of the reference thin film electrode and large reverse direction recombination current due to long electronic transmission distance. When illuminated with Xe-lamp, the sensitivity of the reference thin film electrode increased to 3182 μA mM^-1^ cm^-2^, which was 1.66 times than the sensitivity of the reference thin film electrode in dark condition due to the same reason of IOPCs electrodes with Xe-lamp illumination.

As seen from Fig. 4c, the sensitivity of CuO IOPCs S2 was 2926 μA mM^-1^ cm^-2^ without Xe-lamp illumination and 3559 μA mM^-1^ cm^-2^ with Xe-lamp illumination. There is no significant difference between the sensitivity values of CuO IOPCs S1 and S2, thus indicating that the pore size of the IOPCs does not have much influence on the sensitivity, which is consistent with the results of our previous work^34^.

**Figure 5:**
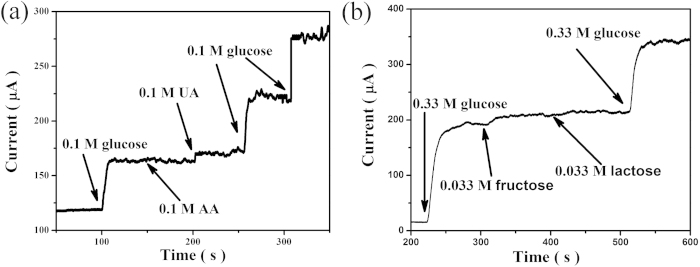
Amperometric response of CdS QDs modified CuO IOPCs FTO electrode with addition of different interfering compounds. (**a**) with successive additions of different interfering species (AA, UA). (**b**) with successive additions of different interfering species (lactose, fructose).

The I−t curves were also measured for CdS QDs modified CuO IOPCs obtained after 3 (Fig. 4d) and 5 (data is not shown) SILAR cycles, and their corresponding calibration curves are shown in Figs. 4e,f. Figs. 4g,h show the calibration curves of amperometric response of three different electrodes with and without Xe-lamp illumination: CuO IOPCs S1, and the two CdS QDs modified CuO IOPCs obtained from 3 and 5 SILAR cycles. The sensitivity values of these three samples were found to be 3417 μA mM^-1^ cm^-2^, 3944 μA mM^-1^ cm^-2^ and 3300 μA mM^-1^ cm^-2^ in the absence of Xe lamp illumination while the sensitivity values were increased to 4065 μA mM^-1^ cm^-2^, 4345 μA mM^-1^ cm^-2^ and 3476 μA mM^-1^ cm^-2^ with Xe-lamp illumination. These results indicate that whether or not the Xe-lamp illumination is used, the sensitivity is increased to the highest value after 3 SILAR cycles of CdS QDs sensitization. On the other hand after 5 SILAR cycles of CdS QDs sensitization, the sensitivity was decreased to lower values than that of the original CuO IOPCs electrode.

The increase in sensitivity after 3 SILAR cycles of CdS QDs sensitization can be attributed to two aspects. Firstly, many surface defects are created during the synthesis of the CuO IOPCs, and these defects could trap unpaired electrons and holes, leading to a decrease in the current. The CdS QDs within interfaces passivate the surface states of CuO IOPCs and block the CuO surface, thus suppressing the recombination of electrons and holes at the interfaces^45^. Secondly, as seen in Fig. 1b, the conduction band of CdS is considerably higher than that of CuO, so the CdS QDs act as a potential barrier to adjust the electric field and potential distribution at the interface, thus suppressing the dark current^46^,^47^. Moreover, when using Xe-lamp illumination, the CdS QDs can absorb light from the visible region of the spectrum and generate new electrons on the surface, improving the current response.

Although the electrode after 5 SILAR cycles of CdS QDs sensitization has larger absorption range and higher absorption intensity, its sensitivity and linear range have a decrease than the electrode after 3 SILAR cycles of CdS QDs sensitization, which can be attributed to the reduction of effective surface area available to provide contact with the glucose molecules, due to the excess deposition of CdS QDs. Thus it appears that 3 SILAR cycles of CdS QDs sensitization is the optimum condition for preparing electrodes, providing the highest sensitivity value of 4345 μA mM^-1^ cm^-2^. In addition, the linear range was obtained over a concentration up to 0.5 mM with a detection limit of 0.15 μM (with signal to noise ratio of 3).

A comparison of CdS QDs modified CuO IOPCs FTO electrodes with some of the other reported metal based non-enzymatic glucose biosensors is presented in Table 1. From the comparison, it can be seen that our new sensor exhibits a good integrative performance characterized by high sensitivity, low detection limit and considerable linear range. In particular, the sensitivity of this sensor is much higher than that of other glucose sensors.

### Selectivity, reproducibility and stability

Anti-interference property is an important parameter for glucose biosensors. Since human blood contains many easily oxidized compounds like ascorbic acid (AA) and uric acid (UA), the influence of other oxidizable interferents must be taken into consideration. As shown in Fig. 5a, the addition of interferents, 0.1 mM of AA and 0.1 mM of UA, gives rise to only negligible current changes, while a significant current response is observed with the subsequent addition of 0.1 mM of glucose. The higher selectivity for glucose over the two interferents can be seen from the current ratios. The current ratio of AA to glucose was determined to be 5.68%, while the current ratio of UA to glucose was 8.33%.

In addition, carbohydrate interferents such as fructose and lactose also present in human blood, thus the selectivity to these carbohydrates were also evaluated. Although the concentration of interfering species in the human blood is 30 to 50 times less than that of glucose^48^, much higher concentrations of the interfering species (with glucose to interfering species ratio of 10) were used in this analysis to ensure the sensitivity. As is clearly seen in Fig. 5b, interfering carbohydrate have little effect compared to glucose. Thus the CdS QDs modified CuO IOPCs FTO electrodes were found to exhibit excellent selectivity in the interference experiments conducted.

**Figure 6:**
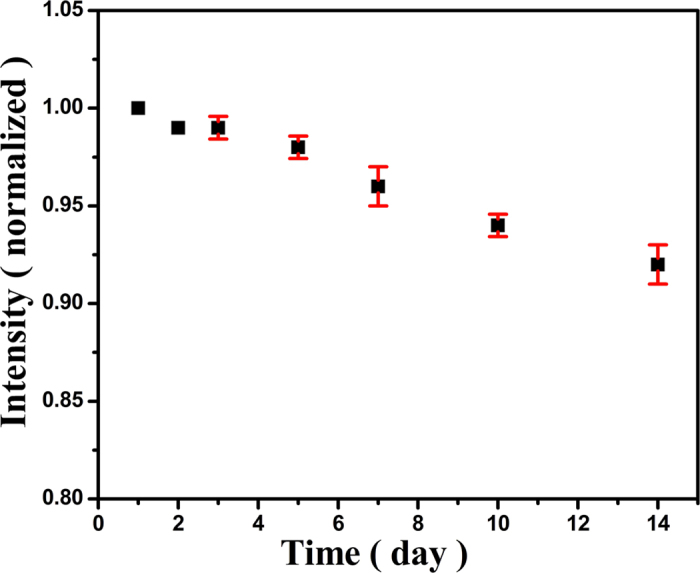
Stability curves of CdS QDs modified CuO IOPCs FTO electrode. The data are average values from three modified electrodes.

To evaluate the reproducibility of the new electrodes, five CdS QDs modified CuO IOPCs FTO electrodes were prepared separately under the same conditions. The relative standard deviations (RSDs) were calculated to be no more than 5.8% (data is not shown), indicating a good reproducibility. The long-term stability of the CdS QDs modified CuO IOPCs FTO electrode was evaluated by placing it in 0.1 M NaOH solution at room temperature for a period of time (Fig. 6). It was found that the CdS QDs modified CuO IOPCs FTO electrode retained 92% of its original current response even after a storage period of 2 weeks, indicating good stability.

The combined results described above indicate that our novel CdS QDs modified CuO IOPCs FTO electrodes exhibit good performance as an electrochemical electrode, including high selectivity, reproducibility and stability.

## Conclusions

In this work, we have synthesized new CuO IOPCs by the sol-gel method using PMMA as a template, and then modified them with CdS quantum dots using a SILAR approach. A series of CdS QDs modified CuO IOPCs FTO electrodes were fabricated using different numbers of SILAR cycles, all of which displayed many attractive analytical properties such as high sensitivity, good stability, good reproducibility, excellent selectivity, as well as quick response. The sensitivity of CuO IOPCs was found to be 4065 μA mM^-1^ cm^-2^, which is likely due to the large surface area and uniform porous distribution within the IOPCs structure. Further increase in sensitivity was attributed to the effect of surface passivation and the protection of the potential barrier of CdS QDs. It was found that 3 SILAR cycles of CdS QDs sensitization was the optimum condition for preparing electrodes, which gave high sensitivity of up to 4345 μA mM^-1^ cm^-2^. Overall, the novel CdS QDs modified CuO IOPCs FTO electrodes demonstrate good performance as non-enzymatic electrochemical and photoelectrochemical glucose biosensors.

## Experimental Section

### Materials

Cupric nitrate trihydrate (Cu (NO_3_)_2_•3H_2_O) and Sodium sulfide nonahydrate (Na_2_S•9H_2_O) were obtained from Tianjin Guangfu Technology development Co., Ltd. in China. Cadmium nitrate tetrahydrate (Cd (NO_3_)_2_•4H_2_O) was obtained from Sinopharm Chemical Reagent Co., Ltd. Methyl methacrylate (MMA), ethanol, glucose and citric acid were purchased from Beijing Chemical Plant (Beijing, P. R. China). All chemicals were analytical grades and used without further purification.

### Synthesis of CuO inverse opal photonic crystals

CuO IOPCs were synthesized by the sol-gel method using the PMMA latex sphere as the colloidal crystal template. Firstly, the monodispersed PMMA spheres with controllable sizes were synthesized using our previously reported method^49^. Fluorine-doped tin oxide (FTO) glass (sheet resistance 30 Ω/cm^2^; Qiseguang Glass Co. Ltd., Dalian) was washed ultrasonically in ace-tone, isopropyl alcohol, and deionized water mixed solution and dried, and then the FTO substrate was soaked in the mixed H_2_SO_4_/H_2_O_2_ aqueous solution with the volume ratio of 3:1 for 1 hour and washed with distilled water to make its surface hydrophilic. Subsequently, the PMMA template was self-assembled through the vertical deposition process. The hydrophilic FTO substrate was submerged into the prepared PMMA colloidal suspension (5% solid content) and kept at 35 °C in an oven for 2 days. The PMMA colloidal spheres were slowly self-organized into highly ordered colloidal arrays on the FTO substrate, driven by surface tension of the liquid in the evaporating process. Following deposition, the photonic crystals were sintered for 40 min at 120 °C to enhance their physical strength.

Cu (NO_3_)_2_•3H_2_O (2.5 g) was dissolved in 10 mL ethanol solution as the copper ions precursor. Then, an appropriate amount of citric acid was added to the solution as chelating agent. The mixture eventually formed a bright blue and transparent solution after stirring for 1 hour. The prepared precursor solution was used to infiltrate into the interstices of the templates under the capillary force from one side of the PMMA templates. After sufficient infiltration, the templates were dried in air overnight. Then, the samples were heated to 500 °C at a rate of 1 °C/min and kept for 3 hours to remove the original PMMA template.

### CdS QDs modified CuO IOPCs FTO electrode

The CuO IOPCs were modified with CdS QDs using a previously reported SILAR route^50^,^51^. In a typical procedure, the CuO IOPCs were immersed in a 20 mL ethanol solution containing 50 mM cadmium nitrate tetrahydrate (Cd (NO_3_)_2_•4H_2_O) for 1 min, to allow Cd^2+^ to adsorb onto the surface of CuO IOPCs. Then, the electrodes were immersed in ethanol solution and dried in a N_2_ stream. The dried electrodes were dipped into a mixture solution of ethanol and water containing 50 mM Sodium sulfide nonahydrate (Na_2_S•9H_2_O) for 1 min, where the adsorbed Cd^2+^ reacted with S^2-^ to form the desired CdS. The electrodes were then rinsed in ethanol for 1 min to remove the excess ions and dried again in a N_2_ stream. This procedure was repeated several times to obtain the desired thickness of CdS.

### Apparatus

The morphology of the samples was inspected using a JEOL JSM-7500F field emission scanning electron microscope (SEM) (Japan) and a JEM-2010 transmission electron microscope (TEM) (Japan) under a working voltage of 200 kV. The high-resolution TEM (HRTEM) images and EDX patterns were measured on a JEOL-2100F high-resolution transmission electron microscope (Japan) with a working voltage of 200 kV. X-ray diffraction (XRD) patterns were conducted on a RigakuD/max 2550 X-ray diffractometer. Electrochemical measurements were performed on a model CHI630D electrochemical analyzer (ChenHua Instruments Co.Ltd., Shanghai, China). Ultraviolet-visible (UV-vis) absorption spectra were measured with a Shimadzu UV-3101PC scanning spectrophotometer. The electrochemical measurements were carried out using a three-electrode system, which employed a platinum wire as the counter electrode, a saturated calomel electrode (SCE) as the reference electrode, and CdS QDs modified CuO IOPCs FTO electrodes as the working electrode.

## Additional Information

**How to cite this article**: Xia, L. *et al.* CdS Quantum Dots Modified CuO Inverse Opal Electrodes for Ultrasensitive Electrochemical and Photoelectrochemical Biosensor. *Sci. Rep.* **5**, 10838; doi: 10.1038/srep10838 (2015).

## Supplementary Material

Supplementary Information

## Figures and Tables

**Table 1:** Comparison of analytical performance of CdS QDs modified CuO IOPCs FTO electrodes with several typical nonenzymatic glucose sensors.

**Electrode**	**Detection range(μM)**	**Detection limit (μM)**	**Sensitivity (μA mM^-1^ cm^-2^)**	**Stability**	**Ref.**
**CuO IOPCs S1**	**0.17 - 1000**	**0.17**	**4065**	**~94% after 14 days**	**this work**
**CuO IOPCs/CdS of 3 SILAR cycles**	**0.15 - 500**	**0.15**	**4345**	**~94% after 14 days**	**this work**
**CuO IOPCs/CdS of 5 SILAR cycles**	**0.19 - 500**	**0.19**	**3476**	**~94% after 14 days**	**this work**
**CuO–SWCNT**	**0.05 - 1800**	**0.005**	**1610**		**Dung et al. (2013)**
**Tantalum oxide honeycomb nanostructures**	**31000**	**0.1**	**160**	**~95% after 15 days**	**Suneesh et al. (2013)**
**CuO nanoparticles-modified carbon nanotube**	**0.4 -1200**	**0.2**	**2596**	**~90% after 30 days**	**Jiang et al. (2010)**
**Ni nanowires**	**0.5 - 7000**	**0.1**	**1043**		**Lu et al. (2009)**
**Au nanoparticles**	**500 - 8000**		**160**		**Kurniawan et al. (2006)**

## References

[b1] Asif M. H. *et al.* Functionalised ZnO-nanorod-based selective electrochemical sensor for intracellular glucose. Biosensors and Bioelectronics, 25, 2205–2211(2010). 20303253 10.1016/j.bios.2010.02.025

[b2] Fang B. *et al.* Silver oxide nanowalls grown on Cu substrate as an enzymeless glucose sensor. ACS Appl. Mater. Interfaces. 12, 2829–2834(2009). 20356163 10.1021/am900576z

[b3] Rakow N. A. & Suslick K. S. A colorimetric sensor array for odour visualization. Nature. 406, 710–714(2000). 10963592 10.1038/35021028

[b4] Cherevko S. & Chung C. H. Gold nanowire array electrode for non-enzymatic voltammetric and amperometric glucose detection. Sensors and Actuators B: Chemical. 142, 216–223(2009).

[b5] El Khatib K. M. & Abdel Hameed R. M. Development of Cu_2_O/Carbon Vulcan XC-72 as non-enzymatic sensor for glucose determination. Biosensors and Bioelectronics. 26, 3542–3548(2011). 21353522 10.1016/j.bios.2011.01.042

[b6] Watanabe T. & Einaga Y. Design and fabrication of nickel microdisk-arrayed diamond electrodes for a non-enzymatic glucose sensor based on control of diffusion profiles. Biosensors and Bioelectronics. 24, 2684–2689(2009). 19261462 10.1016/j.bios.2009.01.041

[b7] Xia C. & Ning W. A novel non-enzymatic electrochemical glucose sensor modified with FeOOH nanowire. Electrochemistry Communications. 12, 1581–1584(2010).

[b8] Park S.; Chung T. D. & Kim H. C. Nonenzymatic Glucose Detection Using Mesoporous Platinum. Anal. Chem. 75, 3046–3049(2003). 12964749 10.1021/ac0263465

[b9] Li Y. *et al.* Hydrogen bubble dynamic template synthesis of porous gold for nonenzymatic electrochemical detection of glucose. Electrochem. Commun. 9, 981–988(2007).

[b10] Yuan B. *et al.* Real time observation of the anodic dissolution of copper in NaCl solution with the digital holography. Electrochemistry Communications. 11, 1373–1376(2009).

[b11] Li Y. & Zhang J. Z. Hydrogen generation from photoelectrochemical water splitting based on nanomaterials. Laser & Photonics Reviews. 4, 517–528(2010).

[b12] Jena B. K. & Raj C. R. Enzyme-Free Amperometric Sensing of Glucose by Using Gold Nanoparticles. Chem. Eur. J. 12, 2702–2708 (2006). 16429473 10.1002/chem.200501051

[b13] Rong L. Q. *et al.* Study of the nonenzymatic glucose sensor based on highly dispersed Pt nanoparticles supported on carbon nanotubes. Talanta. 72, 819–824(2007). 19071692 10.1016/j.talanta.2006.12.037

[b14] Kang X. H. *et al.* A sensitive nonenzymatic glucose sensor in alkaline media with a copper nanocluster/multiwall carbon nanotube-modified glassy carbon electrode. Analytical Biochemistry. 363, 143–150(2007). 17288983 10.1016/j.ab.2007.01.003

[b15] Xu Q. *et al.* Preparation of functionalized copper nanoparticles and fabrication of a glucose sensor. Sensors and Actuators B: Chemical. 114, 379–386(2006).

[b16] Wang W. *et al.* Three-dimensional network films of electrospun copper oxide nanofibers for glucose determination. Biosensors and Bioelectronics. 25, 708–714(2009). 19733046 10.1016/j.bios.2009.08.013

[b17] Mu Y. *et al.* Nano nickel oxide modified non-enzymatic glucose sensors with enhanced sensitivity through an electrochemical process strategy at high potential. Biosensors and Bioelectronics. 26, 2948–2952(2011). 21167705 10.1016/j.bios.2010.11.042

[b18] Springsteen G. & Wang B. Alizarin Red S. as a general optical reporter for studying the binding of boronic acids with carbohydrates. Chem Commun. 17, 1608–1609(2001). 10.1039/b104895n 12240405

[b19] Tharmaraj V. & Pitchumani K. D-Glucose sensing by (E)-(4-((pyren-1-ylmethylene)amino)phenyl) boronic acid via a photoinduced electron transfer (PET) mechanism. Rsc Adv. 29, 11566–11570(2013).

[b20] Neupane L. N *et al.* A dual role of phenylboronic acid as a receptor for carbohydrates as well as a quencher for neighboring pyrene fluorophore. Tetrahedron. 52, 11057–11063(2013).

[b21] Wang Z. J. *et al.* A facile channel for D-glucose detection in aqueous solution. Spectrochim Acta A. 114, 293–297(2013). 10.1016/j.saa.2013.05.089 23778168

[b22] Huang Y. J. *et al.* Glucose sensing via aggregation and the use of “Knock-Out” binding to improve selectivity. J Am Chem Soc. 5, 1700–1703(2013). 23317305 10.1021/ja311442x

[b23] Chen J. *et al.* Temperature dependence of field emission from cupric oxide nanobelt films. Appl. Phys. Lett. 83, 746–748(2003).

[b24] Luque G. L.; Rodriguez M. C. & Rivas G. A. Glucose biosensors based on the immobilization of copper oxide and glucose oxidase within a carbon paste matrix. Talanta. 66, 467–471(2005). 18970008 10.1016/j.talanta.2004.07.019

[b25] Chowdhuri A. *et al.* Response speed of SnO2-based H2S gas sensors with CuO nanoparticles. Appl. Phys. Lett. 84, 1180–1182(2004).

[b26] Sun C. L. *et al.* Ultrasensitive and highly stable nonenzymatic glucose sensor by a CuO/graphene-modified screen-printed carbon electrode integrated with flow-injection analysis. Electrochem. Commun. 30, 91–94(2013).

[b27] Li Y. & Zhang J. Z. Hydrogen generation from photoelectrochemical water splitting based on nanomaterials. Laser Photon. Rev. 4, 517–528(2010).

[b28] Nozik A. J. Nanoscience and Nanostructures for Photovoltaics and Solar Fuels. Nano Lett. 10, 2735–2741(2010). 20597472 10.1021/nl102122x

[b29] Umar A. *et al.* Enzymatic glucose biosensor based on flower-shaped copper oxide nanostructures composed of thin nanosheets. Electrochemistry Communications. 11, 278–281(2009).

[b30] Batchelor-McAuley C. *et al.* The use of copper(II) oxide nanorod bundles for the non-enzymatic voltammetric sensing of carbohydrates and hydrogen peroxide. Sensors and Actuators B: Chemical. 135, 230–235(2008).

[b31] Reitz E. *et al.* CuO Nanospheres Based Nonenzymatic Glucose Sensor. Electroanalysis. 20, 2482–2486(2008).

[b32] Satheesh Babu T. G. & Ramachandran T. Development of highly sensitive non-enzymatic sensor for the selective determination of glucose and fabrication of a working model. Electrochimica Acta. 55, 1612–1618(2010).

[b33] Zhuang Z. *et al.* An improved sensitivity non-enzymatic glucose sensor based on a CuO nanowire modified Cu electrode. Analyst. 133, 126–132(2008). 18087623 10.1039/b712970j

[b34] Xia L. *et al.* Zinc oxide inverse opal electrodes modified by glucose oxidase for electrochemical and photoelectrochemical biosensor. Biosensors and Bioelectronics. 9, 350–357(2014). 24752145 10.1016/j.bios.2014.03.038

[b35] Zhu H. G. *et al.* Synthesis of size-controlled monodisperse manganese carbonate microparticles as templates for uniform polyelectrolyte microcapsule formation. Chem. Mater. 17, 2323(2005).

[b36] Yang P. *et al.* Generalized block copolymer syntheses of large-pore mesoporous metal oxides with semicrystalline frameworks. Nature. 396, 152–155(1998).

[b37] Li X. L.; Jia Y. & Cao A. Y. Tailored Single-Walled Carbon Nanotube−CdS Nanoparticle Hybrids for Tunable Optoelectronic Devices. ACS Nano. 4, 506–512(2010). 20041712 10.1021/nn901757s

[b38] Pan Z. X. *et al.* Highly Efficient Inverted Type-I CdS/CdSe Core/Shell Structure QD-Sensitized Solar Cells. ACS Nano. 6, 3982–3991(2012). 22509717 10.1021/nn300278z

[b39] Ferancov A. *et al.* Electrochemical determination of guanine and adenine by CdS microspheres modified electrode and evaluation of damage to DNA purine bases by UV radiation. Biosensors and Bioelectronics. 26, 314–320(2010). 20829020 10.1016/j.bios.2010.08.026

[b40] Liu Y. *et al.* Magnetic-field induced formation of 1D Fe3O4/C/CdS coaxial nanochains as highly efficient and reusable photocatalysts for water treatment. J. Mater. Chem. 45, 18359–18364(2011).

[b41] Gorer S. & Hodes G. Quantum size effects in the study of chemical solution deposition mechanisms of semiconductor films. J. Phys. Chem. 98, 5338–5346(1994).

[b42] Yochelis S. & Hodes G. Nanocrystalline CdSe Formation by Direct Reaction between Cd Ions and Selenosulfate Solution. Chem. Mater. 16, 2740–2744(2004).

[b43] Cheng C. *et al.* Strontium titanate nanoparticles as the photoanode for CdS quantum dot sensitized solar cells. RSC Adv. 5, 4844–4852 (2015).

[b44] Jiang L. C. & Zhang W. D. A highly sensitive nonenzymatic glucose sensor based on CuO nanoparticles-modified carbon nanotube electrode. Biosensors and Bioelectronics. 25, 1402–1407(2010). 19942424 10.1016/j.bios.2009.10.038

[b45] Li W. L. *et al.* Highly sensitive and selective photoelectrochemical biosensor platform for polybrominated diphenyl ether detection using the quantum dots sensitized three-dimensional, macroporous ZnO nanosheet photoelectrode. Biosensors and Bioelectronics. 61, 209–214(2014). 24892782 10.1016/j.bios.2014.04.058

[b46] Lee H. *et al.* Multilayered Semiconductor (CdS/CdSe/ZnS)-Sensitized TiO_2_ Mesoporous Solar Cells: All Prepared by Successive Ionic Layer Adsorption and Reaction Processes. Chem. Mater. 22, 5636–5643(2010).

[b47] Xu G. P. *et al.* Effect of ZnS and CdS coating on the photovoltaic properties of CuInS_2_-sensitized photoelectrodes. J. Mater. Chem. 22, 4890–4896(2012).

[b48] Cao F. *et al.* Highly sensitive nonenzymatic glucose sensor based on electrospun copper oxide–doped nickel oxide composite microfibers. Talanta. 86, 214–220 (2011). 22063533 10.1016/j.talanta.2011.09.003

[b49] Zhu Y. S. *et al.* Inhibited Long-Scale Energy Transfer in Dysprosium Doped Yttrium Vanadate Inverse Opal. J. Phys. Chem. C. 116, 2297–2302(2012).

[b50] Baker D. R. & Kamat P. V. Photosensitization of TiO_2_ Nanostructures with CdS Quantum Dots: Particulate versus Tubular Support Architectures. Adv. Funct. Mater. 19, 805–811(2009).

[b51] Cheng C. W. *et al.* Quantum-Dot-Sensitized TiO_2_ Inverse Opals for Photoelectrochemical Hydrogen Generation. Small. 8, 37–42(2012). 22009604 10.1002/smll.201101660

