# Supplementary material for: CdS quantum dots modified CuO inverse opal electrodes for ultrasensitive electrochemical and photoelectrochemical biosensor
Source: Sci Rep. 2015 Jun 4;5:10838. doi: 10.1038/srep10838 (PMC4455289; doi:10.1038/srep10838)
Supplement: Supplementary Information [file srep10838-s1.doc]

**CdS Quantum Dots Modified CuO Inverse Opal Electrodes for Ultrasensitive Electrochemical and Photoelectrochemical Biosensor**

Lei Xia, Lin Xu, Jian Song, Ru Xu, Dali Liu*, Biao Dong, Hongwei Song*

State Key Laboratory on Integrated Optoelectronics, College of Electronic Science and Engineering, Jilin University, 2699 Qianjin Street, Changchun 130012, China

Corresponding to:

Prof. Dali Liu E-Mail: [ldl@jlu.edu.cn](mailto:ldl@jlu.edu.cn) Fax: 86-0431-85168270

Prof. Hongwei Song E-Mail: [songhw@jlu.edu.cn](mailto:songhw@jlu.edu.cn) Fax: 86-0431-85155129

Supporting information


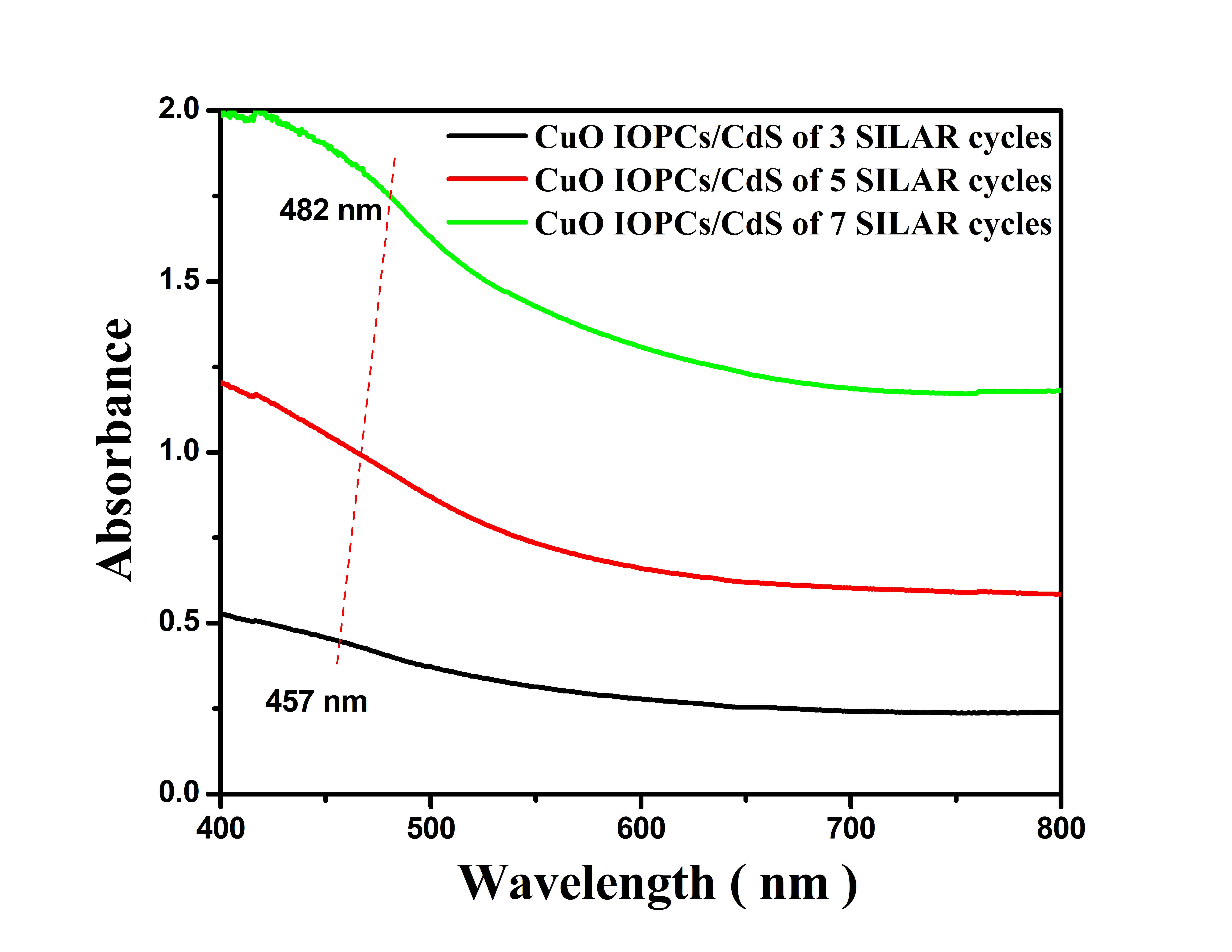


Figure S1. Absorption spectra of different electrodes of Figure 3b, using CuO IOPCs S1 as the reference


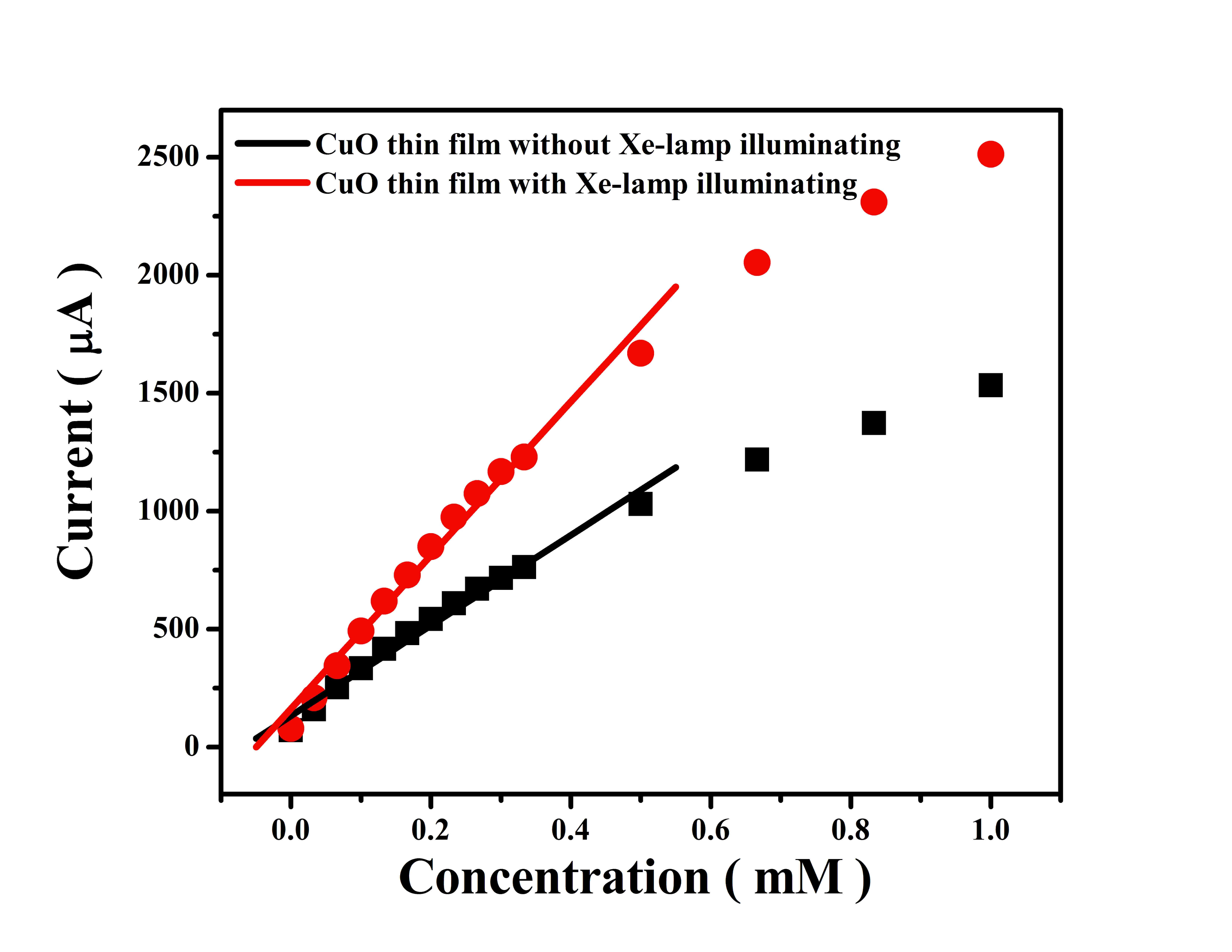


Figure S2. Calibration curves of amperometric response of CuO film FTO electrode to successive addition of glucose at an applied potential of 0.7 V.


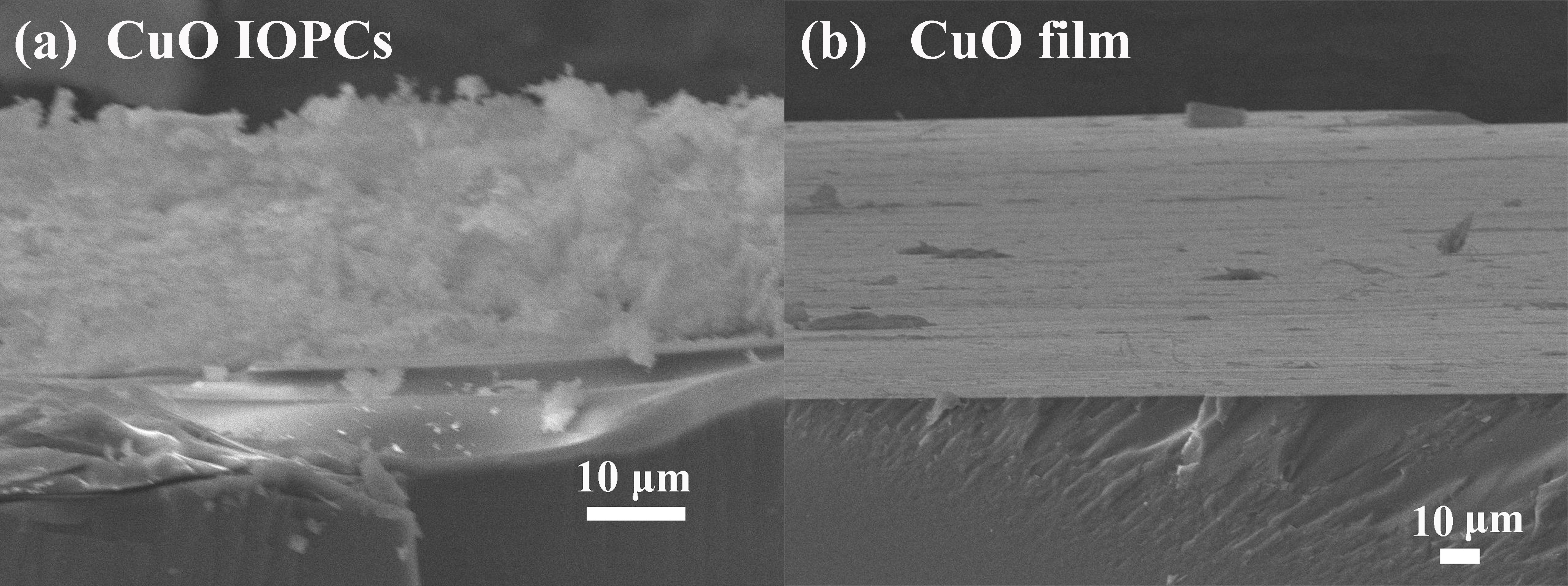


Figure S3. SEM images of cross-section view of CuO IOPCs FTO electrode (a) and CuO film FTO electrode (b).
